# Comprehensive Analysis of Autophagy-Associated lncRNAs Reveal Potential Prognostic Prediction in Pancreatic Cancer

**DOI:** 10.3389/fonc.2021.596573

**Published:** 2021-05-26

**Authors:** Guangyu Chen, Gang Yang, Junyu Long, Jinshou Yang, Cheng Qin, Wenhao Luo, Jiangdong Qiu, Fangyu Zhao, Lei You, Taiping Zhang, Yupei Zhao

**Affiliations:** ^1^ Department of General Surgery, Peking Union Medical College Hospital, Chinese Academy of Medical Sciences and Peking Union Medical College, Beijing, China; ^2^ Department of Liver Surgery, Peking Union Medical College Hospital, Chinese Academy of Medical Sciences and Peking Union Medical College, Beijing, China; ^3^ Clinical Immunology Center, Chinese Academy of Medical Sciences and Peking Union Medical College, Beijing, China

**Keywords:** pancreatic cancer, long noncoding RNA, autophagy, prognosis, TCGA, ICGC

## Abstract

Pancreatic cancer (PC) is a highly malignant tumor in the digestive system. Both long noncoding RNAs (lncRNAs) and autophagy play vital roles in the development and progress of PC. Here, we constructed a prognostic risk score system based on the expression profile of autophagy-associated lncRNAs for prognostic prediction in PC patients. Firstly, we extracted the expression profile of lncRNA and clinical information from The Cancer Genome Atlas (TCGA) and International Cancer Genome Consortium (ICGC) databases. The autophagy-associated genes were from The Human Autophagy Database. Through Cox regression and survival analysis, we screened out seven autophagy-associated lncRNAs and built the risk score system in which the patients with PC were distinguished into high- and low-risk groups in both training and validation datasets. PCA plot displayed distinct discrimination, and risk score system displayed independently predictive value for PC patient survival time by multivariate Cox regression. Then, we built a lncRNA and mRNA co-expression network *via* Cytoscape and Sankey diagram. Finally, we analyzed the function of lncRNAs in high- and low-risk groups by gene set enrichment analysis (GSEA). The results showed that autophagy and metabolism might make significant effects on PC patients of low-risk groups. Taken together, our study provides a new insight to understand the role of autophagy-associated lncRNAs and finds novel therapeutic and prognostic targets in PC.

## Introduction

Pancreatic cancer (PC) is a poorly prognostic malignant tumor. Its incidence and mortality rank second and fifth in digestive system tumors in the United States and China separately, of which the five-year survival rate is about 9% (1, 2). Current treatments cannot significantly improve the prognosis of PC patients; meanwhile, the development of pancreatic cancer treatment is relatively slow compared to other tumors, so surgery still represents the most effective treatment to cure resectable pancreatic cancer. Due to the lack of diagnosis at the early stage and the highly malignant characteristics of PC, PC patients frequently exhibit lymph node metastasis and local invasion when the diagnosis is made, leading to approximately 80% of patients losing surgical chances (3). Therefore, the exploration of more effective innovative targets for pancreatic cancer is urgent and necessary.

Autophagy is the homeostatic mechanism through a membrane-mediated process that delivers cytoplasmic organelles and proteins to lysosomes for degradation. There is growing evidence that the level of autophagy can be responded by intracellular and extracellular stresses, such as ER stress, oxidative stress, hypoxia, nutrient shortage, etc., thereby involving tumor progression (4). In pancreatic cancer, autophagy plays a significant tumorigenic role in keeping cancer cell survival and promoting metabolism (5). Hydroxychloroquine (HCQ), which can inhibit autophagy combined with Gemcitabine, is currently being tested in many clinical trials. Consequently, researching new biomarkers related to autophagy to improve early diagnosis and assess prognosis is a promising avenue for PC patients.

Long noncoding RNAs (lncRNAs) mostly have no protein-coding potential of which transcripts are longer than 200 nucleotides. lncRNA can affect different functions at an epigenetic, transcriptional, and post-transcriptional level, and play a vital role in regulating cancer cell behaviors and autophagy (6). There is a recent study indicated that downregulated lncRNA LINC00160 suppressed autophagy and drug resistance in hepatocellular carcinoma by regulating miR-132-targeted PIK3R3 (7). Zhang et al. elaborated lncRNA PVT1 induced cytoprotective autophagy and promoted growth *via* sponging to miR-20a-5p and regulating ULK1 both *in vitro* and *in vivo* in pancreatic ductal adenocarcinoma (8). Another study also demonstrated that silencing lncRNA LINC00160 facilitated autophagy and apoptosis of pancreatic cancer cells (9). Considering that several lncRNAs may influence cancer behaviors through mediating autophagy, it is crucial to explore autophagy-associated lncRNAs to predict the prognosis of PC patients.

In our current study, we analyze the relationship between autophagy-associated lncRNA profiles and clinical information in 178 PC patients from The Cancer Genome Atlas (TCGA) database. The survival analysis showed that seven lncRNAs (AC245041.2, LINC02257, AC006504.8, AC012306.2, AC125494.2, FLVCR1-DT, and AC005332.6) were prognostic biomarkers for patients with PC. Then, the seven lncRNAs were used to develop a risk score system after Cox regression analysis. Finally, we constructed a prognostic signature that can be applied to independently predict the prognostic of PC patients. These candidate autophagy-associated lncRNAs may become the potential prognostic prediction for PC.

## Materials and Methods

### Sample Datasets

The RNA-seq data and clinical information of PC patients were downloaded from the TCGA data portal (https://portal.gdc.cancer.gov/) and ICGC (https://icgc.org) databases, respectively. Then, we transformed the RNA sequence data to the lncRNAs and mRNA (protein coding) based on the annotated gene IDs in the Ensembl project. Because the data were extracted from the public database, there was no requirement for ethics committee approval.

### Identification of Autophagy-Related lncRNAs

The autophagy gene list was obtained from The Human Autophagy Database (http://www.autophagy.lu/index.html), employing Pearson correlation analysis to screen the relationship between the lncRNAs and autophagy-related genes. An absolute value of correlation coefficient > 0.4 (|R|>0.4) and P < 0.05 were considered statistically significant. Based on the above standard, the autophagy-related lncRNAs were filtrated for subsequent analysis.

### Survival Analysis

Kaplan–Meier (K-M) survival analyses of the autophagy-associated lncRNA were performed using the survival package in R. The patients were classified into high expression and low expression groups using optimal cut-off values determined by the survminer R package (Version:0.4.3). Log-rank P < 0.05 was considered statistical significant.

### Construction of Co-Expression Network and Function Analysis

To better understand the relation between lncRNAs and mRNAs, the lncRNA-mRNA co-expression network was visualized by Cytoscape software (http://www.cytoscape.org/). To investigate the functions of these lncRNAs, the co-expression of mRNAs was analyzed by gene ontology (GO) terms enrichment including biological process, molecular function, and cellular component. A P value of < 0.05 was statistically significant.

### Construction of the Risk Score System

Firstly, we used the univariate Cox regression analysis to confirm prognostic autophagy-associated lncRNAs. These lncRNAs were significantly associated (P < 0.001) with overall survival (OS). Then, multivariate Cox regression analysis was employed to screen ultimate autophagy-associated lncRNAs and predict the regression coefficients (β) of the model. Finally, a prognosis risk score system based on seven genes was established. Risk score = (β1 × expression level of AC006504.8) + (β2 × expression level of FLVCR1-DT) + (β3 × expression level of AC012306.2) + (β4 × expression level of AC125494.2) + (β5 × expression level of AC005332.6) + (β6 × expression level of AC245041.2) + (β7 ×expression level of LINC02257). Based on an optimal cutoff value, all PC patients were divided into low‐ and high‐risk groups. To estimate the predictive capacities of the risk score system by constructing Kaplan–Meier survival curves and receiver operating characteristic (ROC) curves.

### Gene Set Enrichment Analysis (GSEA)

We use GSEA software (http://www.broadinstitute.org/gsea) to identify the underlying different functions between the high- and low-risk groups. The annotated gene sets c5.all.v7.1.symbols.gmt was chosen for the reference gene sets. Enriched gene sets were considered to be statistically significant by a nominal P value < 0.05 that was set as the cut-off criteria.

### Cell Culture

The human pancreatic ductal epithelium cell line HPNE and PC cell line PANC-1 were purchased from the ATCC. The cells were cultured at 37°C in a humidified atmosphere containing 5% CO2 with high glucose DMEM supplemented with 10% fetal serum. The culture medium and supplements were purchased from HyClone (Northbrook, IL, USA).

### RNA Extraction and qRT-PCR

Total RNA was extracted by Trizol reagent (Invitrogen, 15596018). RNAs were reverse transcribed utilizing PrimeScriptTM RT Master Mix (TaKaRa, RR036A). qRT-PCR was performed using the TB Green Fast qPCR Mix (TaKaRa, RR430S). The primer sequences were used as follows: AC245041.2: Forward 5′-TCCAGACAAGCAGGATGTGG-3′, Reverse 5′-AGAGGTTTATAGAGGGAGATGGGA-3′; LINC02257: Forward 5′-GAGACCTTTCACCGGGCTTT-3′, Reverse 5′-GCTTCTTGCTGTGTGTTTCCC-3′; AC006504.8: Forward 5′-GAACACAAGCCCGTTAGCA-3′, Reverse 5′-AGTGGGGTATGGGTAATAGGATAG-3′; AC012306.2: Forward 5′- TGCTCCCTTACCCTTATGGC-3′, Reverse 5′-GAGCATGGGGCCGTATTTTA-3′; AC125494.2: Forward 5′-ATCTCCAACCCTGACATTCGG-3′, Reverse 5′-CAGGGAAGAACAGAAGCCGAT-3′; FLVCR1-DT: Forward 5′-TAACGCCAGAAAGTGTTCCAGT-3′, Reverse 5′-CTGCTCCATCATAGCCCGTC-3′; AC005332.6: Forward 5′-CTCATGTGCTTCTTCTGGGCTT-3′, Reverse 5′-TGGCACTCTAATGTTTGCTGACT-3′; GAPDH: Forward 5′-GTATCGTGGAAGGACTCATGAC-3′, Reverse 5′- ACCACCTTCTTGATGTCATCAT-3′.

## Results

### Identification of Seven Prognostic Autophagy-Associated lncRNAs in PC Patients

We extracted a total of 14,142 lncRNAs expression data of tumors from PC tissues in the TCGA database. Two hundred thirty-two autophagy-associated genes were selected from The Human Autophagy Database. We then utilized Pearson correlation analysis to screen the co-expression relationship between the lncRNAs and autophagy-associated genes with the criteria of |R| > 0.4 and P < 0.05. Conclusively, 1,234 autophagy-associated lncRNAs were screened. To identify autophagy-associated lncRNAs related to prognosis, we selected the above filtered autophagy-associated lncRNAs by univariate Cox regression analysis and found that 29 lncRNAs were significantly related to the PC patients’ overall survival(OS) (**Table 1**). Then, we performed multivariate Cox regression analysis to screen the optimal prognostic lncRNAs. Finally, a total of seven lncRNAs were identified (**Table 2**). Among these lncRNAs, AC245041.2 and LINC02257 were risk factors (HR > 1), and AC006504.8, AC012306.2, AC125494.2, FLVCR1-DT, and AC005332.6 were protective factors (HR < 1).

**Table 1:** Univariate cox regression analysis of prognostic autophagy-associated lncRNAs.

Gene	P value	HR	Lower 95% CI	Upper 95% CI
LINC01004	0.00013	0.669899	0.545603	0.822512
AC005696.1	5.49E-05	0.350479	0.210584	0.58331
AC006504.8	3.04E-05	0.442255	0.301411	0.648913
FLVCR1-DT	0.000122	0.350297	0.205107	0.598264
AC036176.1	0.000401	0.508103	0.349241	0.739229
AC012306.2	1.67E-05	0.513715	0.37932	0.695727
U62317.1	0.000738	1.118986	1.048265	1.194478
AC127024.5	2.28E-05	0.516408	0.380336	0.701163
AL022328.4	8.62E-05	0.316545	0.178267	0.562082
AL513165.1	0.000402	0.793017	0.697425	0.901711
AC090114.2	5.71E-05	0.421068	0.276314	0.641655
AC125494.2	7.25E-05	0.272869	0.143661	0.518286
AC064836.3	0.000284	0.599275	0.454493	0.790178
AC006449.6	0.000149	0.445464	0.293297	0.676579
AC142472.1	0.00021	0.374297	0.222604	0.629361
AC005332.6	0.000205	0.850786	0.781215	0.926553
LINC01089	0.000315	0.763742	0.659587	0.884345
AL022328.1	0.000578	0.573619	0.417996	0.787182
ST20-AS1	7.66E-05	0.19611	0.087474	0.439665
AL122010.1	3.35E-05	0.538231	0.401665	0.72123
AC245041.2	7.99E-05	1.222149	1.106203	1.350247
AC005332.5	0.000223	0.571746	0.424898	0.769345
AC005332.3	7.90E-06	0.735942	0.643327	0.841889
AL358472.2	3.22E-05	0.28793	0.160095	0.517842
AC020765.2	0.000811	0.397367	0.231539	0.681963
PTOV1-AS2	0.000333	0.822028	0.738571	0.914916
AC145207.5	8.70E-05	0.347442	0.204919	0.58909
LINC01705	8.72E-05	1.108166	1.052746	1.166504
LINC02257	6.07E-06	1.525904	1.270616	1.832484

HR, hazard ratio; CI, confidence interval.

**Table 2:** Multivariate cox regression analysis of prognostic autophagy- associated gene.

Gene	Coef	HR	Lower 95% CI	Upper 95% CI
AC006504.8	-0.3765	0.6862	0.4339	1.0853
FLVCR1-DT	-0.5525	0.5755	0.3283	1.0090
AC012306.2	-0.4120	0.6623	0.4618	0.9500
AC005332.6	-0.1040	0.9012	0.8152	0.9964
AC125494.2	-0.5192	0.5950	0.3221	1.0991
AC245041.2	0.2093	1.2328	1.0870	1.3982
LINC02257	0.2490	1.2828	1.0257	1.6043

Coef, coefficient; HR, hazard ratio; CI, confidence interval.

### Survival Analysis of Autophagy-Associated lncRNAs

**Figure 1:**
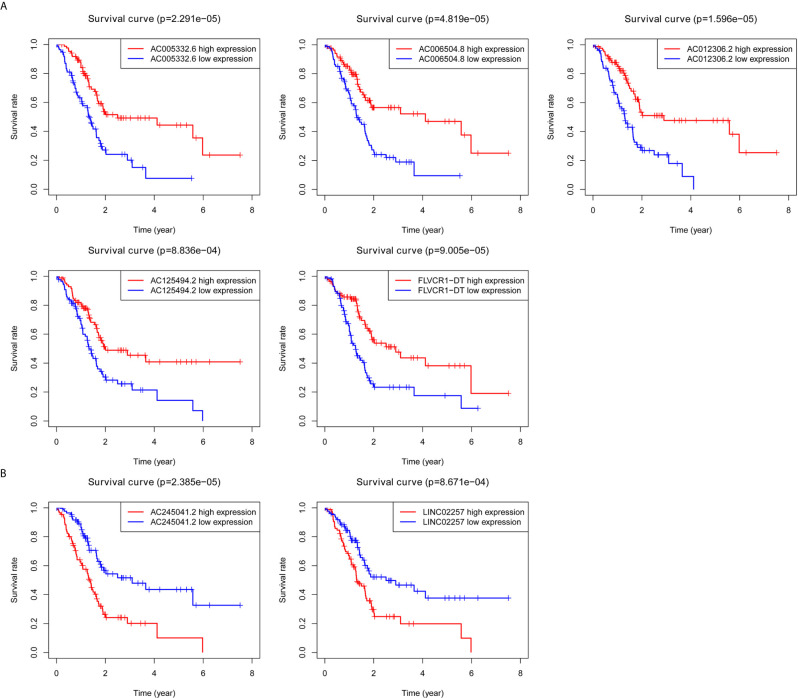
Survival analysis for autophagy-associated lncRNAs. Kaplan–Meier survival curves for autophagy-associated lncRNAs that were positively **(A)** or negatively **(B)** related to OS in PC patients.

Kaplan–Meier survival analyses and log‐rank tests for each autophagy-associated lncRNA were performed to evaluate the prognostic characteristics of patients with PC. K-M survival curves of the seven autophagy-associated lncRNAs are shown that the high expression of AC005332.6, AC006504.8, AC012306.2, AC125494.2, and FLVCR1-DT were positively correlated with the longer overall survival of patients with PC (p < 0.01), indicating protective impacts of these lncRNAs in PC development (**Figure 1A**). Furthermore, the high expression of AC245041.2 and LINC02257 was correlated with a short survival time (p < 0.01), which meant that these lncRNAs could play a carcinogenic role in PC (**Figure 1B**).

### Construction and Validation of the Risk Score Evaluation System of the Autophagy-Associated lncRNAs

**Figure 2:**
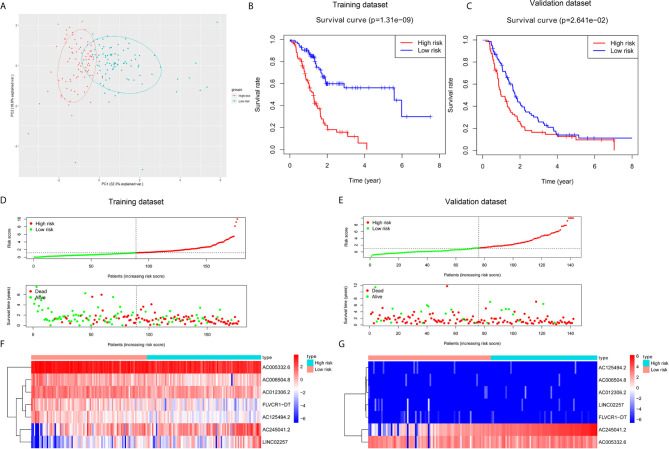
Construction and validation of a prognostic risk score system for PC. **(A)** Principal component analysis (PCA) based on the high- and low-risk group indicated two significantly distinct patterns. **(B, C)** Kaplan–Meier survival curves showed that PC patients with high-risk scores suffered shorter survival time than those with low-risk scores in the training and validation dataset. **(D, E)** Risk score level between high-risk and low-risk groups. A scatter plot showed the distribution of the survival status and survival time in high- and low-risk groups. **(F, G)** Heatmap of the screened autophagy-associated lncRNAs expression profiles with different risk groups in the training and validation dataset.

Based on seven lncRNAs that were significantly correlated with overall survival, the autophagy-associated lncRNA signature was constructed to predict the outcome of PC patients. The final risk score formula was as follows: Risk score = (-0.3765 × expression level of AC006504.8) + (-0.5525 × expression level of FLVCR1-DT) + (-0.4120 × expression level of AC012306.2) + (-0.5192 × expression level of AC125494.2) + (-0.1040 × expression level of AC005332.6) + (0.2476 × expression level of AC245041.2) + (0.2490 ×expression level of LINC02257). With the above formula, the risk scores of more than 0.1199 were classified into the high-risk group (88 patients); meanwhile, those with less than the cutoff point belonged to the low-risk group (89 patients). The principal components analysis (PCA) showed that the high-risk and low-risk groups were divided into two obvious distribution patterns, which implied that autophagy made distinctly different effects in two groups (**Figure 2A**). Based on K‐M survival analyses, the patients with low-risk scores had longer survival time than those with high-risk scores; OS had statistical significance between the two subgroups (**Figure 2B**). We used scatter diagrams to show the risk scores, survival status, and survival time of each PC patient (**Figure 2D**). The results demonstrated that the PC patient’s survival time and rate gradually deteriorated with increasing risk scores. Moreover, lncRNAs’ expression profiles were shown by a heatmap plot (**Figure 2F**). We extracted the PACA-CA cohort from the ICGC database to further validated the prognostic stability of the risk evaluation system. Then, we utilized the same formula and cutoff value above to classify the PC patients into low-risk and high-risk groups according to the TCGA dataset. Similarly, the patients from the high-risk group (66 patients) had a lower survival time than the low-risk group (76 patients) (**Figure 2C**). Moreover, the scatter diagrams and a heatmap plot of lncRNAs expression profiles were also shown (**Figures 2E, G**).

### The Autophagy-Related lncRNA Signature Was an Independent Prognostic Factor

**Figure 3:**
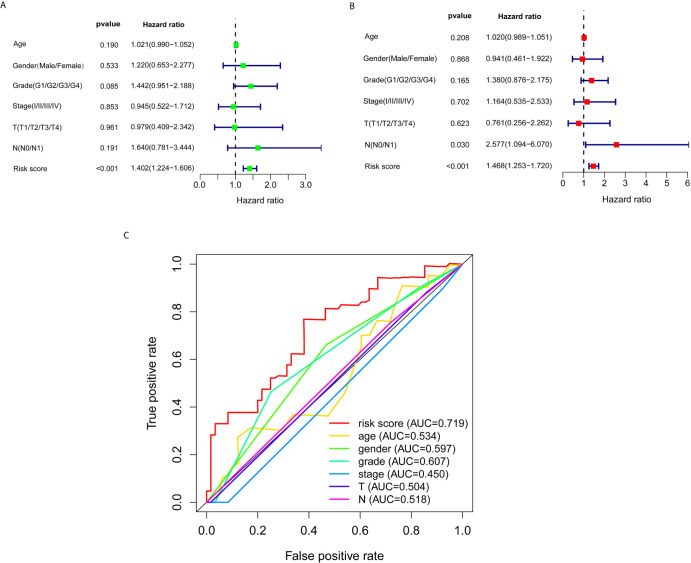
Effects of the risk score and clinical information on the prognosis of PC patients. **(A)** To identify the relationship between the risk score or clinical information with OS by univariate Cox analyses. **(B)** To identify the relationship between the risk score or clinical information with OS by multivariate Cox analyses. **(C)** ROC curves analysis of OS for the prognosis risk score and the classical clinical parameters.

Subsequently, univariate and multivariate Cox regression analyses were conducted to screen potential biomarkers correlated with OS and total clinical information (**Figures 3A, B**). The results showed that only the prognostic value of the risk score was statistically significant. Finally, the receiver operating characteristic (ROC) curve analysis and the AUC value were utilized to assess the prediction accuracy of the above results. The AUC value of risk score based on expression profiles of autophagy-associated lncRNAs was equal to 0.719, which was much higher than age curve (AUC = 0.534), gender curve (AUC= 0.597), grade curve (AUC= 0.607), stage curve (AUC=0.450), T stage curve (AUC=0.504), and N stage curve (AUC= 0.518) (**Figure 3C**), suggesting that the risk score was superior to traditional clinical indicators. Thus, the risk score evaluation system derived from the expression levels of the seven lncRNAs was the unique independent prognostic indicator of survival time for PC patients.

### Construction of lncRNA–mRNA Network and Enrichment Analysis of GO and KEGG

**Figure 4:**
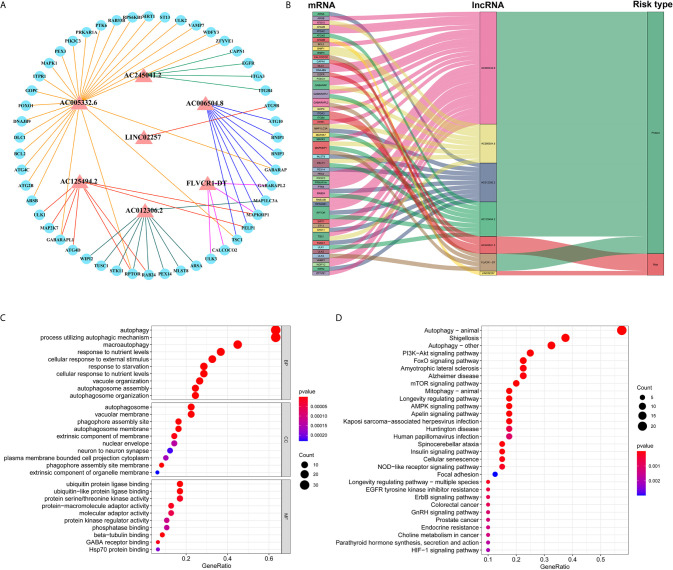
Construction of lncRNA–mRNA co-expression network and GO and KEGG enrichment analysis. **(A)** The lncRNA–mRNA network between seven autophagy-associated lncRNAs and relevant mRNAs. Red triangles indicate autophagy-associated lncRNAs. Blue circles indicate mRNAs. The line represents a co-expression relationship between the lncRNA and the mRNA. **(B)** A Sankey diagram showed the co-occurrences of lncRNAs, mRNAs, and characters according to the risk score. **(C)** GO analysis of biological processes, cell components, and molecular functions based on the co-expressed mRNAs. **(D)** KEGG analysis showed the significantly enriched pathways based on the co-expressed mRNAs.

Based on the abovementioned analysis, to better understand the potential effect of lncRNAs on mRNAs in PC, we built the lncRNA-mRNA network and used Cytoscape and Sankey diagram to visualize the network. We constructed the lncRNA-mRNA co-expression network using the screened seven autophagy-associated lncRNAs with Pearson correlation analysis (|R| > 0.4 and P < 0.05). A total of 61 lncRNA-mRNA pairs were filtrated and the correlation between lncRNAs, mRNAs, and risk score groups by the Sankey diagram (**Figures 4A, B**). Furthermore, GO enrichment analysis was performed to clarify the biological processes, cellular components, and molecular function of mRNAs, which were identified from the lncRNA-mRNA co-expression network. As shown in bubble plot revealing top 10 GO terms, We found that the foremost biological processes were “autophagy”, “process utilizing autophagic mechanism”, and “macroautophagy”; the top three cellular components were “autophagosome”, “vacuolar membrane”, and “phagophore assembly site”; the top three molecular functions were “ubiquitin protein ligase binding”, “ubiquitin-like protein ligase binding”, and “protein serine/threonine kinase activity” (**Figure 4C**). KRGG enrichment analysis was shown that autophagy, shigellosis, PI3K-Akt signaling pathway, and FoxO signaling pathway were the top four significantly enriched pathways (**Figure 4D**).

### Gene Set Enrichment Analysis (GSEA)

**Figure 5:**
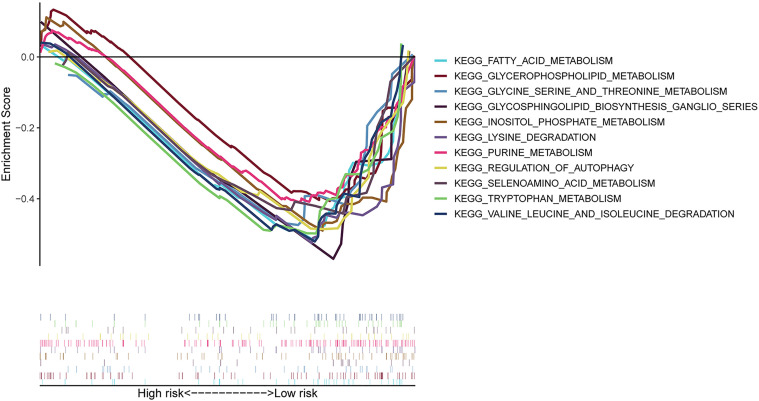
GSEA between high- and low-risk groups based on the identified risk score system. GSEA result indicated that the autophagy-related and metabolic pathways were significantly enriched in the low-risk group.

We carried out the GSEA of the PC samples based on the TCGA to identify the biological pathways associated with the high-risk group and low-risk group. We did not discover a significantly enriched pathway in the high-risk group; moreover, the low-risk group was most significantly enriched for “neuroactive ligand receptor interaction”, “tryptophan metabolism”, “lysine degradation”, “glycosphingolipid biosynthesis ganglio series”, “regulation of autophagy”, “inositol phosphate metabolism”, “glycerophospholipid metabolism”, “fatty acid metabolism”, etc (**Figure 5**). In summary, the GSEA analysis results elaborated that the low-risk score group was closely correlated with autophagy and metabolism. These KEGG data may provide valuable targets to treat for PC.

### Expression of Seven Autophagy-Associated lncRNAs in HPNE and PANC-1 Cells

**Figure 6:**
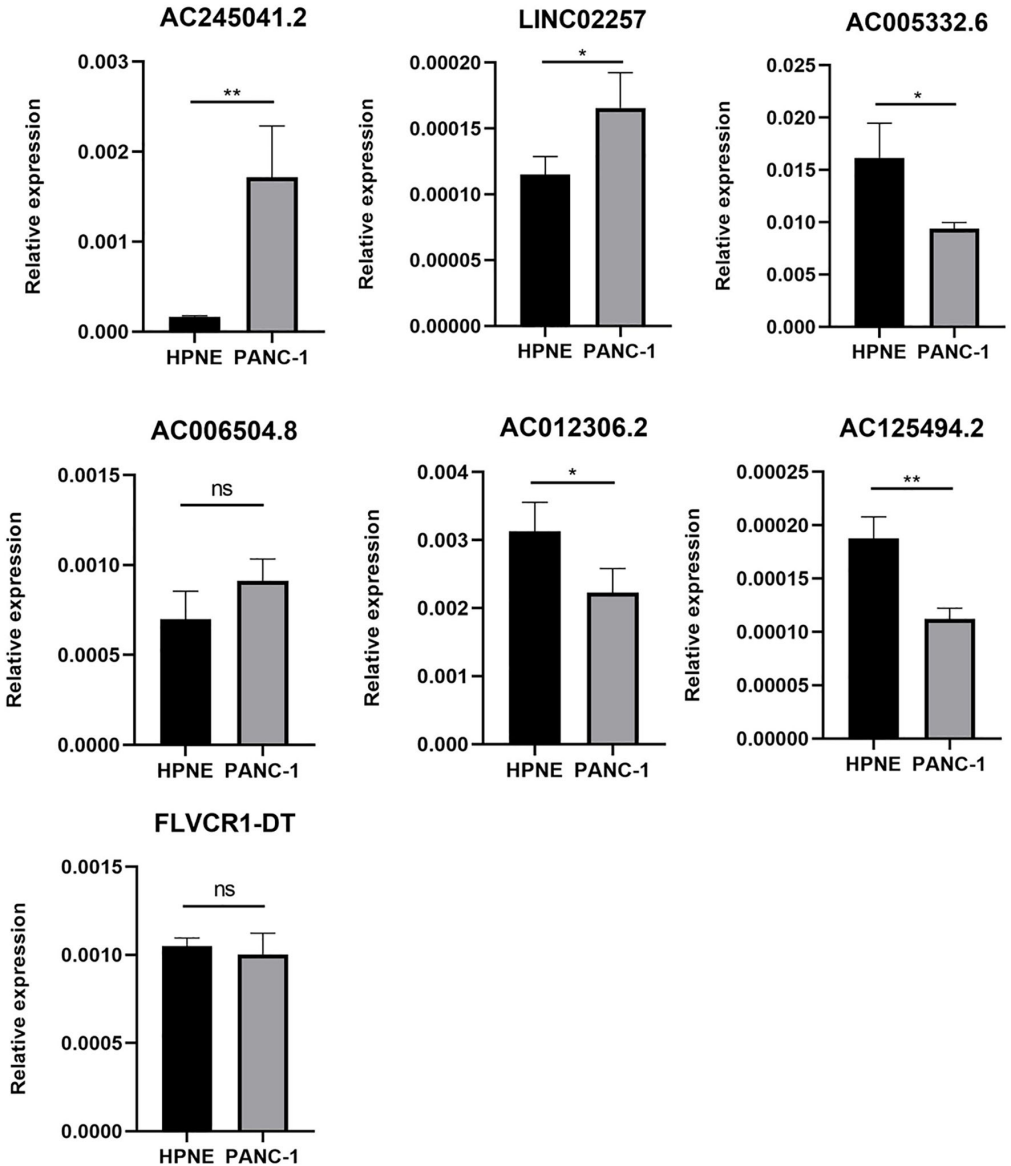
Expression of 7 lncRNAs in HPNE and PANC-1 cells. The qRT-PCR result showed that AC245041.2 and LINC02257 were high-expressed in PANC-1. AC005332.6, AC012306.2, AC125494.2 were low-expressed in PANC-1. The expression of FLVCR1-DT and AC006504.8 was not statistically different between PANC-1 and HPNE. *P < 0.05, **P < 0.01, NS, no statistically significant.

As is evident from **Figures 1A, B**, AC005332.6, AC006504.8, AC012306.2, AC125494.2, and FLVCR1-DT may protective factors; moreover, AC245041.2 and LINC02257 were carcinogenic factors in PC. Therefore, we analyzed the expression of these lncRNAs in the PC cell line PANC-1 and normal human pancreatic ductal epithelium cell line HPNE. Our results indicated that AC245041.2 and LINC02257 were high-expressed in PANC-1. The expression of FLVCR1-DT and AC006504.8 was not statistically different between PANC-1 and HPNE, while the low expression of AC005332.6, AC012306.2, and AC125494.2 were in PANC-1 (**Figure 6**).

## Discussion

PC is a highly malignant digestive cancer with the lowest five-year survival rate in various types of cancer and is predicted to be the second leading cause of cancer-related death in the U.S. by 2030 (10). Most patients with PC cannot be diagnosed early; meanwhile, carbohydrate antigen (CA19–9) as a conventional diagnostic biomarker is not applied to specifically and sensitively diagnose the PC patients (11, 12). Only a small part of PC patients can be treated by traditional surgery, and a large number of patients suffer from tumor recurrence and progression. Consequently, the identification of PC regulatory factors has become the focus of recent clinical and basic research. To detect PC early and provide new therapeutic options are of great importance. Currently, LncRNAs have been found to play vital roles in PC and are indispensable for carcinogenetic function, especially autophagy (13, 14). An increasing amount of evidence has shown that autophagy‐associated lncRNAs make great effects on the occurrence, development, and prognosis of cancer (15).

To better understand the roles of lncRNAs involved with autophagy in the occurrence of PC. Firstly, we analyzed the expression profiles of lncRNAs in PC patients from the TCGA database. We used Pearson analysis to identify the co-expression relationship between lncRNAs and autophagy-related genes in The Human Autophagy Database. Seven autophagy-associated lncRNAs significantly correlated with survival were selected to build the risk score system *via* the multivariate Cox regression analysis. The high- and low-risk patients can be distinguished according to the median risk score, and PCA analysis displayed a significantly distinct distribution between these two groups. Notably, low-risk patients had a better prognosis than patients in the high-risk group. To assess the potency of the risk score model, we utilized the data from the ICGC database as a validation dataset and got the same result. The AUC value that was calculated from the ROC curves indicated that the risk score had considerable prognostic accuracy for PC patients. Furthermore, the risk score was considered as an independent prognostic factor by univariate and multivariate Cox regression analysis.

lncRNAs play an important role in affecting mRNA expression through regulating histone modifications, DNA methylation, and acting as miRNA sponges or precursors of miRNAs, involving the process of transcriptional regulation, post-transcriptional regulation, and epigenetic regulation (16). To elucidate the probable roles of the seven autophagy-associated lncRNAs in PC, the lncRNA-mRNA co-expression network was constructed. The GO and KEGG enrichment analysis was subsequently performed on these mRNAs related to screened lncRNAs, and the results showed that the top enriched GO and KEGG terms were significantly correlated with autophagy. Subsequently, as shown in the GSEA result, we observed that the low-risk group enriched many pathways about lipid, amino acid metabolism, and autophagy, suggesting that metabolism and autophagy were greatly associated with the PC patients of the low-risk score. The above results have shown that the specific autophagy mechanisms were closely related to PC progression.

Besides, there are some limitations that exist in our study. We built the co-expression network between the lncRNAs and mRNA, but how the lncRNAs make specific effects on mRNA was unknown. Furthermore, the autophagy‐associated lncRNAs were only detected in PANC-1 and HPNE. The specific molecular mechanisms have not been verified in the experiments. Furthermore, in the existing studies, there are only a few bioinformatic analyses about some lncRNAs in our risk score system. Chen J et al. found that AC245041.2 was a risk factor in clear cell renal cell carcinoma (ccRCC) and high expression of AC245041.2 was associated with a poor outcome for patients with ccRCC (17). LINC02257 was found to correlate with the prognosis of patients with colorectal cancer and was a risk factor (18). Besides, AC006504.8 was a risk factor in cholangiocarcinoma (19). Concerning the filtered lncRNAs, we need exploratory experiments to prove the functions deeply.

In conclusion, we successfully established the risk score system based on the seven autophagy‐associated lncRNAs; meanwhile, it was an independent prognostic factor in PC patients. This approach enhances the prediction accuracy for target lncRNAs, and these autophagy‐associated lncRNAs might be of great significance for the prediction of prognosis and therapeutic markers for PC patients.

## Data Availability Statement

Publicly available datasets were analyzed in this study. This data can be found here: The Human Autophagy Database (http://www.autophagy.lu/), The Cancer Genome Atlas (https://portal.gdc.cancer.gov/) and International Cancer Genome Consortium (https://icgc.org).

## Ethics Statement

The studies involving human participants were reviewed and approved by The Ethics Committee of Peking Union Medical College Hospital. The patients/participants provided their written informed consent to participate in this study.

## Author Contributions

GC analyzed the data and wrote the manuscript. TZ and YZ conceived the study and obtained financial support. LY and JL applied guiding suggestion, GY, JY, CQ, WL, JQ, and FZ prepared the dataset. All authors contributed to the article and approved the submitted version.

## Funding

This study was supported by grants from the National Natural Science Foundation of China (No. 81772639, No.81802475, No.81972258, No.81974376); Natural Science Foundation of Beijing (No. 7192157); CAMS Innovation Fund for Medical Sciences (CIFMS) (No.2016-I2M-1-001); National Key R&D Program of China (2018YFE0118600); Non-profit Central Research Institute Fund of Chinese Academy of Medical Sciences (2019XK320001). The funder bodies were not involved in the study design, collection, analysis, interpretation of data, the writing of this article or the decision to submit it for publication.

## Conflict of Interest

The authors declare that the research was conducted in the absence of any commercial or financial relationships that could be construed as a potential conflict of interest.
